# Metachronous breast cancer in a male with previous history of liposarcoma: A case report from Syria

**DOI:** 10.1016/j.amsu.2021.103151

**Published:** 2021-12-04

**Authors:** Rand Tarrab, Rami Sabouni, Lana Jarad, Nour Mansour, Maher S. Saifo

**Affiliations:** a Faculty of Medicine, Damascus University, Damascus, Syria; b Department of Oncology, AL-Bairouni University Hospital, Damascus University, Damascus, Syria

**Keywords:** Breast cancer, Carcinoma, Invasive ductal carcinoma, Liposarcoma, Male, Metachronous, Multiple primary cancers

## Abstract

**Introduction:**

Multiple primary cancers (MPC) are defined as the occurrence of two or more non-related cancers. The acquiring of male breast cancer (MBC) as secondary cancer in a sequence of MPC is extremely rare. Only one case of breast cancer following liposarcoma (LP) was previously reported in a female patient. We report the first case of MBC following LP.

**Case presentation:**

A non-smoker male patient with a history of a well-differentiated liposarcoma was treated surgically and with radiotherapy 14 years ago with no signs of recurrence. The patient presented with a left breast mass; The excisional biopsy showed poorly differentiated grade III invasive ductal carcinoma. The patient underwent a mastectomy with axillary node resection and the final diagnosis was invasive ductal carcinoma stage IIA [T:2, N:0, M:0]. The tumor markers reported; Positive Estrogen Receptor (ER+), negative Progesterone Receptor (PR-), and negative Human Epidermal Receptor (HER-). He received eight sessions of chemotherapy with Docetaxel and 16 fractions of radiotherapy. The follow-up showed no signs of recurrence.

**Discussion:**

Despite the rarity of diagnosis MBC as a second primary. Studies have found a relation between different types of breast cancer in male patients, and further, a relation was also found between MBC and lymphoma. While no studies that link MBC and LP were previously reported.

**Conclusion:**

We found that acquiring a treated LP would not affect the MBC prognosis or its response to treatment, yet further studies are needed to confirm this outcome.

## Highlights

• Multiple primary cancers occur in 2.4–17% of all people.
• The diagnosis of male breast cancer as a second primary is rare.
• Having male breast cancer following liposarcoma is even rarer.
• Well treated liposarcoma would not affect male breast cancer prognosis.

## Abbreviation

MPC: multiple primary cancers
MBC: male breast cancer
LP: liposarcoma
BC: breast cancer
ER: Estrogen Receptor
PR: Progesterone Receptor
HER: Human Epidermal Receptor

## 1 Introduction

Multiple primary cancers (MPC) are defined as the occurrence of two or more non-related cancers [1], with a prevalence between 2.4-

% [2]. The recent improvements in diagnostic tests and screening enabled physicians to detect higher numbers of cancer cases; this might be the reason for the increasing numbers of diagnosed MPCs in recent decades [[3], [4], [5], [6]].

The diagnosis of male breast cancer (MBC) is rare, and having it as a second primary cancer is even rarer. Studies found that there is an association between different types of cancer in male patients. However, the information about the association between MBC and non-breast cancers is still not clear due to its rarity [7].

Having MBC in a sequence of MPC following liposarcoma (LP) is a rare condition, and only one case was found in the literature for a 57-year-old woman [8]. We report the first case of male breast cancer as a second primary cancer following LP. This case report has been reported in line with the SCARE Criteria [9].

## 2 Case presentation

**Fig. 1:**
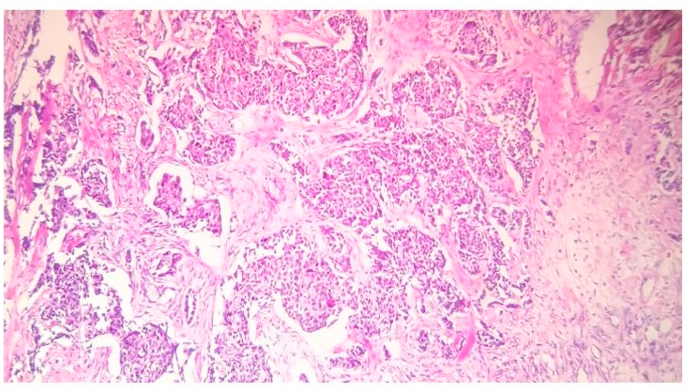
Histological examination: Fig. 1 legend: Invasive ductal carcinoma H&E.

**Fig. 2:**
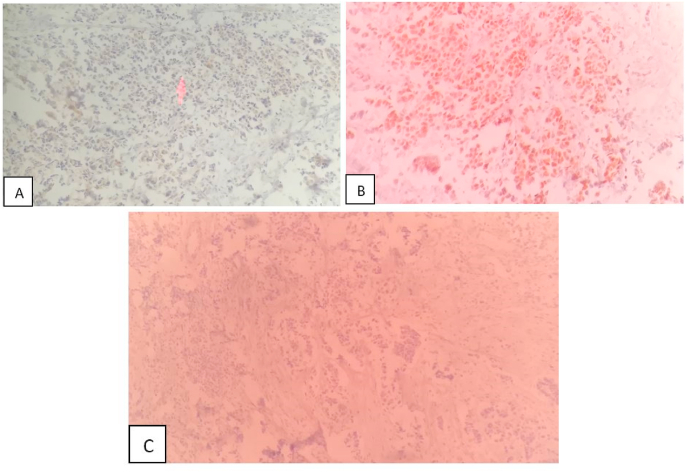
Hormonal receptors tests: Fig. 2 legend: A: HER2 (−); B:ER (+); C: PR (−).

**Fig. 3:**
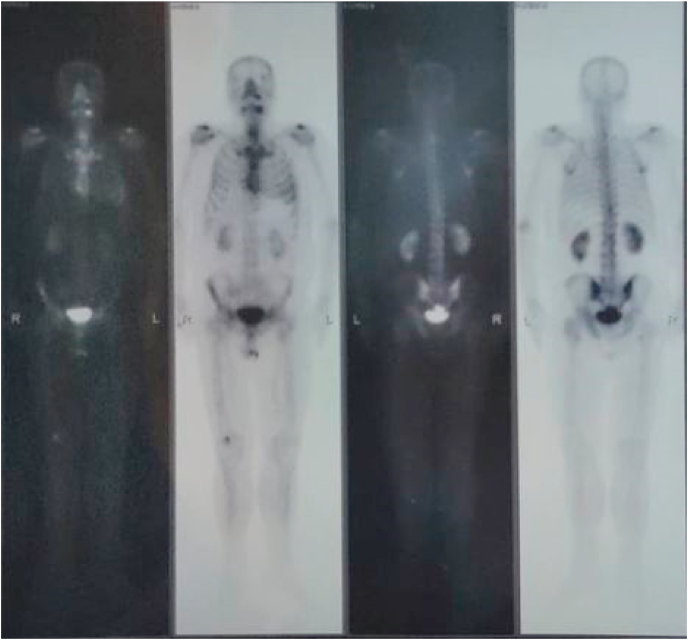
PET scan Fig. 3 legend: The PET scan shows no signs of recurrence.

A 57-year-old retired male presented in 2019 to Al-Bairouni Hospital complaining of a mass in his left breast. He is a non-smoker, with a history of grade (I) LP in the right thigh root 14 years ago which was treated surgically along with radiotherapy of the right thigh, with no signs of recurrence. He also had well-controlled diabetes. The physical examination showed a left breast mass with a suspicious abnormality on Ultrasound that measured 32 mm. The computed tomography scan (CT) showed left breast infiltration with no signs of metastases. The patient underwent an excisional biopsy which revealed a poorly differentiated grade III invasive ductal carcinoma (Fig. 1). Then he underwent a mastectomy in 2019 with axillary node resection. The final diagnosis based on the histological findings was invasive ductal carcinoma stage IIA [T:2, N:0, M:0]. Hormonal receptors tests showed the following: positive Estrogen Receptor (ER+), negative Progesterone Receptor (PR-), and negative Human Epidermal Receptor (HER-) (Fig. 2). He received eight cycles of chemotherapy with Docetaxel and 16 sessions of radiation to the chest wall; The last one was on the fourth of October 2020. A positron emission tomography (PET) scan six months after the surgery showed no signs of recurrence (Fig. 3).

## 3 Discussion

MPC can occur synchronously or metachronously depending on the time interval between the diagnosis of each tumor [10]. Synchronous MPC occurs when the tumor is diagnosed within two or six months according to different recommendation criteria [11,12]. In our case, the tumors were metachronous since the breast cancer was diagnosed 14 years after the diagnosis of LP.

Many risk factors were linked to the increased incidence of MPC including; Genetic susceptibility and familial cancer syndromes as well as environmental exposures (e.g., smoking), hormonal factors, immune deficiency, and infections [13]. Cancer survivors were reported to have twice the probability of developing a second primary cancer which was linked to the long-term effects of treatments, and the increased following-up and monitoring of cancer survivors helped in increasing the number of detected cases [2,3]. Our patient was a cancer survivor and he had no risk factors for breast cancer.

MBC is considered rare and occurs in 0.5–1% [14], and having MBC as a second primary is even rarer. However, A study covered 464 males who were diagnosed with breast cancer as their second MPC found that the most common primary cancer in those men was: prostate (42.9%), colorectal (10.6%), urinary (8.4%), breast (7.5%), lymphoma (6.9%), and melanoma (5.0%) [15]. Also, the risk of developing a second MBC after primary breast cancer was previously reported [7,16]. However, limited information is known regarding the risk of having MBC after non-breast cancer [7]. Studies suggest that the treatments used in the first cancer may be considered as a risk factor for developing the subsequent MBC [7]. The median age of diagnostic MBC as a second primary was reported to be in the 8th decade, and the most common histology was invasive ductal carcinoma [7].

Although a statistically significant association (p = 0.014) between MBC and lymphoma was previously found, no association between MBC and LP was assessed due to the limited information [7]. After searching the literature, we found only one case reporting the occurrence of invasive breast cancer ER+/HER2-, and LP of the thigh in a 57-years old woman. This presentation that mimics our case might raise the suspicion of a possible link between BC and LP [8]. Nevertheless, there is not enough evidence to build this theory. That's been said, further investigation is needed to see if there is a real association or it is just a coincidence.

The main obstacle in dealing with MPC patients is treating both tumors without causing interaction that would worsen the outcome [16]. Nevertheless, the treatment plan depends on the clinical situation of each patient [17]. In our case, we did not have such an obstacle since the MBC was diagnosed 14 years after the LP, and within these 14 years, no signs of recurrence were found. However, the main question here is: does having a previously treated LP change the response to BC treatment? As far as we know, having a treated LP would not affect the prognosis of the tumor response to treatment.

## 4 Conclusion

The incidence of MPC is becoming more common and the need for clear treatment guidelines is becoming an urgent need. We found that having a treated LP would not affect the MBC response to treatment.

## Funding statement

No funding was applied.

## Guarantor

Dr. Maher S. Saifo.

## Provenance and peer review

Not commissioned, externally peer-reviewed.

## Ethical approval

N/A.

## Author contribution

RT, RS, LJ, NM drafted the manuscript. MS supervised the project. All authors have read and approved the final manuscript.

## Registration of research studies

1. Name of the registry: N/A
2. Unique Identifying number or registration ID:
3. Hyperlink to your specific registration (must be publicly accessible and will be checked):

## Annals of medicine and surgery

The following information is required for submission. Please note that failure to respond to these questions/statements will mean your submission will be returned. If you have nothing to declare in any of these categories then this should be stated.

## Consent

Written informed consent was obtained from the patient for publication of this case report and accompanying images. A copy of the written consent is available for review by the Editor-in-Chief of this journal on request.

## Declaration of competing interest

No conflict of interest.
